# Clinical Syndromes and Genetic Screening Strategies of Pheochromocytoma and Paraganglioma

**DOI:** 10.15586/jkcvhl.2018.113

**Published:** 2018-12-27

**Authors:** Peihua Liu, Minghao Li, Xiao Guan, Anze Yu, Qiao Xiao, Cikui Wang, Yixi Hu, Feizhou Zhu, Hongling Yin, Xiaoping Yi, Longfei Liu

**Affiliations:** 1 Department of Urology, Xiangya Hospital, Central South University, Changsha, China; 2 Department of Biochemistry and Molecular Biology, School of Life Sciences, Central South University, Changsha, China; 3 Department of Pathology, Xiangya Hospital, Central South University, Changsha, China; 4 Department of Radiology, Xiangya Hospital, Central South University, Changsha, China

**Keywords:** multiple endocrine neoplasia, von Hippel–Lindau syndrome, neurofibromatosis-1, pheochromocytoma, paraganglioma

## Abstract

Pheochromocytomas (PCCs) are rare neuroendocrine tumors that originate from chromaffin cells of the adrenal medulla, and paragangliomas (PGLs) are extra-adrenal pheochromocytomas. These can be mainly found in clinical syndromes including multiple endocrine neoplasia (MEN), von Hippel–Lindau (VHL) syndrome, neurofibromatosis-1 (NF-1) and familial paraganglioma (FPGL). PCCs and PGLs are thought to have the highest degree of heritability among human tumors, and it has been estimated that 60% of the patients have genetic abnormalities. This review provides an overview of the clinical syndrome and the genetic screening strategies of PCCs and PGLs. Comprehensive screening principles and strategies, along with specific screening based on clinical symptoms, biochemical tests and immunohistochemistry, are discussed.

## Introduction

Pheochromocytomas (PCCs) and paragangliomas (PGLs), with an incidence of 2–8 per million population per year (1, 2), are rare neuroendocrine tumors that originate from chromaffin cells within or outside the adrenal medulla. With the recent recognition of the metastatic potential of PCC and PGL, risk stratification incorporating the metastatic potential, rather than just differentiation of benign or malignant, is recommended (3). PCC and PGL are thought to have the highest heritability among human tumors (4). Up to 40% of PCC and PGL are attributed to germline mutations, and overall, germline and somatic mutations can be present in 60% of PCC and PGL (5). Hereditary PCC and PGL (HPCC/PGL) are usually associated with neoplasm syndromes including multiple endocrine neoplasia (MEN 1 and MEN 2), von Hippel–Lindau (VHL), neurofibromatosis-1 (NF-1), and familial paraganglioma (FPGL). Apart from these, some catecholamine-secreting syndromes such as Sturge–Weber syndrome, tuberous sclerosis complex, ataxia-telangiectasia syndrome, and Carney Trilogy can be related to PCC and PGL (6). To date, there are 29 genes known to be related to PCC and PGL and the exploration of new genes is far from over (7). Genetic screening is useful to identify carriers of the pathogenic mutations of PCC and PGL. With the development of high-throughput screening technologies and comprehensive screening strategies, significant progress has been made in the field. This review provides an overview of the clinical syndromes and genetic screening strategies for PCC and PGL.

## Clinical Syndromes of PCC/PGL

**Figure 1:**
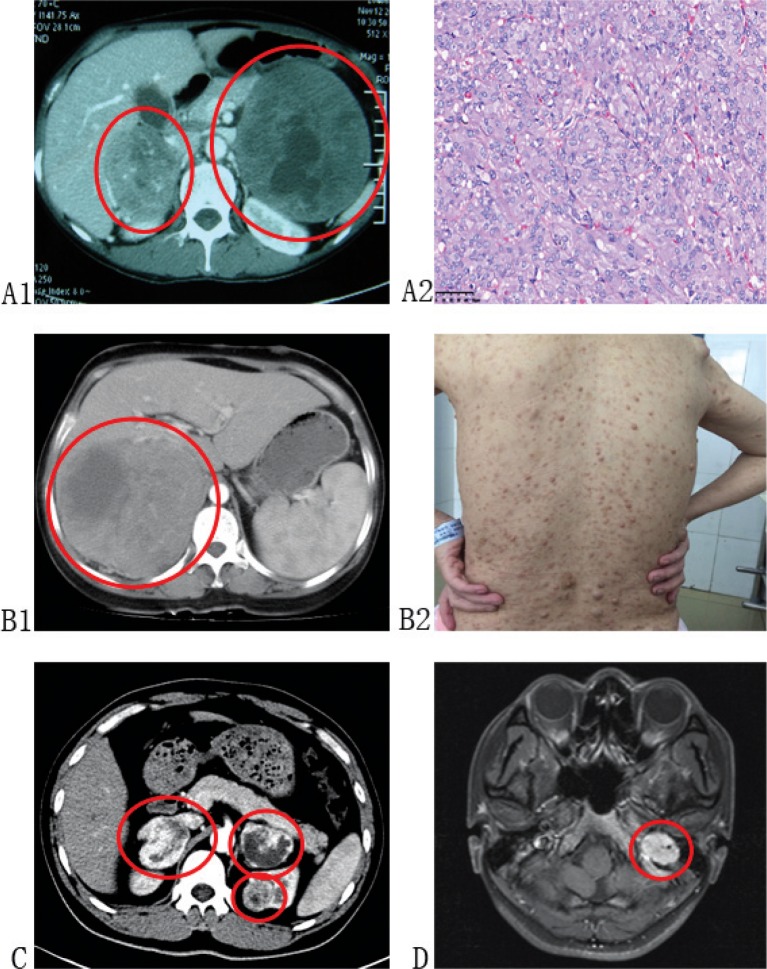
Clinical presentations of PCC/PGL-related syndromes. (A1 and A2): Patient diagnosed with MEN-2 presented with bilateral PCC and MTC. (B1 and B2): Patient diagnosed with NF-1 presented with PCC of right adrenal and multiple neurofibromas on her body. (C): Patient diagnosed with VHL presented with bilateral PCC and left ccRCC. (D): Patient diagnosed with FPGL presented with PGL in her left foramina jugulare, and genetic testing identified germline SDHB mutation of her and her families.

PCC and PGL are considered to be part of clinical syndromes when it comes to hereditary form. An understanding of these syndromes may help to raise awareness of PCC and PGL systematically. The PCC/PGL-related syndromes are summarized in Table 1, and the clinical presentations of PCC/PGL-related syndromes are depicted in Figure 1.

**Table 1:** PCC/PGL-related syndromes

Clinical syndrome	Subtype	Main manifestations	Mutation gene	Genetic nature
MEN 1		PHPT, gastrinomas, benign insulinomas, anterior pituitary tumors, and PCC	*MEN 1*	Tumor suppressor
MEN 2	MEN 2A	MTC, PCC, hyperthyroidism, and amyloidosis of the skin	*RET*	Proto oncogene
	MEN 2B	MTC, PCC, hyperthyroidism, multiple mucosal neuromas, and Marfan-like syndrome	*RET*	Proto oncogene
	FMTC	HMTC	*RET*	Proto oncogene
VHL	Type 1A	Hemangioblastoma in retina and central nervous system, multiple abdominal neoplasms and cysts, ccRCC, and no PCC	*VHL*	Tumor suppressor
	Type 1B	Hemangioblastoma in retina and central nervous system, multiple abdominal neoplasms and cysts, and no ccRCC or PCC	*VHL*	Tumor suppressor
	Type 2A	PCC, hemangioblastoma in retina or central nervous system, and no ccRCC	*VHL*	Tumor suppressor
	Type 2B	PCC, hemangioblastoma in retina or central nervous system, ccRCC, endocrine neoplasia, and pancreatic cyst	*VHL*	Tumor suppressor
	Type 2C	Only PCC	*VHL*	Tumor suppressor
NF-1		Caft-au-lait macules, neurofibromas, freckling in the axillary or inguinal region, optic glioma, lisch nodules, osseous lesion, and PCC	*NF-1*	Tumor suppressor
FPGL	FPGL-1	Multiple head and neck PGLs	*SDHD*	Tumor suppressor
	FPGL-2	Head and neck PGLs	*SDHAF2*	Tumor suppressor
	FPGL-3	Solitary head and neck PGLs	*SDHC*	Tumor suppressor
	FPGL-4	The abdomen, pelvis, and mediastinum PGLs	*SDHB*	Tumor suppressor
	FPGL-5	Leigh’s syndrome	*SDHA*	Tumor suppressor
TMEM127		PCC and rare renal cancers	*TMEM127*	Tumor suppressor
MAX		PCC/PGL	*MAX*	Tumor suppressor
FH		Cutaneous and uterine leiomyomas, type 2 papillary renal carcinoma, and rare PCC/PGL	*FH*	Tumor suppressor

PHPT, primary hyperparathyroidism; PCC, pheochromocytoma; MTC, medullary thyroid carcinoma; HMTC, hereditary medullary thyroid carcinoma; ccRCC, clear-cell renal cell carcinoma; PGL, paraganglioma.

### Multiple Endocrine Neoplasia 2

MEN 2 is an autosomal-dominant inheritance syndrome characterized by the presence of diseases such as medullary thyroid carcinoma (MTC), PCC, and hyperthyroidism (8). MEN 2 has three subtypes: MEN 2A, MEN 2B, and familial MTC (FMTC). Mutations of the proto-oncogene *RET*, located at chromosome 10q11.2, are responsible for the pathogenesis, and nearly 90% of MEN 2 is caused by single point mutation. The *RET* gene encodes tyrosine kinase receptor (TK receptor), which binds to the glial cell line-derived neurotrophic factor (GDNF) and modulates the targets downstream. There are 21 exons in the *RET* gene, and mutations in exons 10, 11, 13, 14, and 15 are responsible for the pathogenesis of MEN 2A (9, 10). According to Tang et al. (11), the incidence rate of MEN 2A is 1/25,000 and FMTC is 1.54/1,000,000; MEN 2B is the rarest form afflicting approximately 5% of the MEN 2 patients. All MEN 2 patients will suffer from MTC at an average age of 20 (10–30 years), about 50% cases of MEN 2 will contract PCC/PGL and 20–30% cases will develop parathyroid tumors (12). However, PCC caused by the mutation of *RET* has a relative low risk of becoming a malignant tumor, and the characteristic clinical manifestation is pathogenesis in both adrenal glands, which easily relapse. MTC typically occurs in MEN 2A patients during adulthood, in MEN 2B patients in childhood, and in FMTC patients in midlife. Some MEN 2A patients will develop amyloidosis of the skin. The clinical manifestations of MEN 2B are similar to MEN 2A with some considerably various characteristics such as multiple mucosal neuromas and Marfan-like syndrome (13).

### Multiple Endocrine Neoplasia 1

MEN 1 is also an autosomal-dominant disease caused by inactivating mutation of *MEN 1* tumor suppressor gene that is located on chromosome 11q13. This gene consists of 10 exons that encode a 610-amino acid suppressor protein, menin, whose main function is maintaining DNA stability and gene regulation. The prevalence of MEN1 is approximately 1/30,000 (14). Neuroendocrine tumors of parathyroid gland, pancreas, and pituitary gland are more common in MEN1, with occasional tumors of the adrenal, thymus, and bronchi (15). Therefore, when these lesions appear on the parathyroid gland, pancreas, and pituitary gland, MEN 1 should be considered. Biochemical tests for parathyroid hormone (PTH), calcium concentrations, glucagonomas, prolactin (PRL), and growth hormone (GH) can assist in the diagnosis of MEN 1. The most common clinical manifestation of MEN 1 is primary hyperparathyroidism (PHPT), which can occur in 90% of patients. The prevalence of MEN 1 patients who exhibit gastro-entero-pancreatic (GEP) tract lesions, such as gastrinomas and benign insulinomas, varies from 30% to 70% (16). The occurrence of anterior pituitary tumors in MEN 1 ranges between 10% and 60% (16).

### Von Hippel–Lindau Syndrome

VHL is an autosomal-dominant inheritance syndrome caused by the germline mutations of VHL tumor suppressor gene. The VHL gene, which has three exons, is located in chromosome 3p25. The clinical manifestations of VHL syndrome include hemangioblastomas of retina and central nervous system, multiple neoplasms (clear cell carcinoma, multiple endocrine neoplasia, and pheochromocytoma), and cysts (multiple cysts in kidney and pancreas) (17). VHL contains a significant variation in phenotype and an age-related penetrance (18). The incidence rate of VHL is 1/36,000 (19), and there is an extremely high penetrance which reaches 95% before 60 years old. Although there has been a significant development in the recognition of molecular biology of VHL and advances in management strategies, the average survival age of VHL patients is mere 49 years. According to the possibility of suffering from PCC, VHL can be separated into two types: type 1 and type 2 (20). Type 1 VHL can also be separated into two subtypes: type 1A and type 1B, based on the probability of developing renal cell carcinoma. Type 1A patients will develop ccRCC without suffering from PCC. Therefore, VHL patients who suffer from PCC are recognized as Type 2. Type 2 VHL syndrome can also be classified into type 2A (hemangioblastoma, PCC, no ccRCC), type 2B (hemangioblastoma, PCC, and ccRCC), and type 2C (the only clinical manifestation is PCC). The VHL gene encodes the VHL protein (pVHL), which has a wide range of functions (21), the most important being the degradation of hypoxia inducible factors (HIFs). Without degradation, HIFs can be translocated to the nucleus and initiate transcription of multiple target genes, which can promote cell proliferation, angiogenesis, erythropoiesis, and anaerobe metabolism (22, 23).

### Neurofibromatosis-1

NF-1 is also an autosomal-dominant disorder caused by mutation of the NF-1 gene that is located at chromosome 17q11.2, whose main function is to inhibit cell proliferation by converting RAS protein into its inactive form (6). Mutation can cause tumors to grow out of control, most commonly on peripheral nerves. NF-1 has a variety of clinical manifestations: caft-au-lait macules, neurofibromas, freckling in the axillary or inguinal region, optic glioma, lisch nodules, osseous lesion, and PCC. The diagnosis of NF-1 is usually based on its clinical presentations. The National Institutes of Health (NIH) suggest that if two or more of the above manifestations are found in someone, NF-1 should be considered by the clinician, and several examination measures may help the diagnosis, for example, CT or MRI for the detection of optic gliomas and plexiform neurofibromas, and biopsy for histologic confirmation. It is important to choose different examination measures to detect different clinical manifestations. There are also several variants of NF-1: segmental neurofibromatosis in which the lesions are confined to one or more well-circumscribed regions; spinal neurofibromatosis in which the tumors are mostly on the spinal nerve roots; NF-Noonan syndrome that is characterized by microsomia, webbed neck, and congenital heart disease; and Watson syndrome that is characterized by pulmonic stenosis, caft-au-lait macules, and decreased IQ (6, 24).

### Other Catecholamine-Secreting Syndromes

Catecholamine-secreting tumors, although rare, can be found in patients with other neurocutaneous syndromes such as Sturge–Weber syndrome (encephalotrigeminal angiomatosis—a congenital neurological and skin disorder), tuberous sclerosis complex (a multisystem genetic disease), ataxia-telangiectasia syndrome (a neurodegenerative, autosomal recessive disease causing severe disability), and Carney–Trilogy (gastrointestinal stromal tumor, pulmonary chondroma, and extra-adrenal paraganglioma) (25).

## SDH Mutations and Familial Paragangliomas

Mutations in the succinate dehydrogenase (SDH, mitochondrial complex II) and its subunit genes *(SDHA, SDHB, SDHC, SDHD,* and *SDHAF2*) can lead to FPGLs. The SDH is a part of both the electron transport chain and the tricarboxylic acid (TCA) cycle (26). It is worth noting that mutation in *SDHB* is possibly related to malignancy and poor prognosis (27). Mutations in *SDHD* and *SDHAF2* are frequently found in head and neck PGLs in the paternal branch. *SDHA-*, *SDHAF2-,* and *SDHC*-related PGLs are infrequent.

### FPGL-1

Mutations in *SDHD* are responsible for FPGL-1 (23–25), which are inherited in an autosomal-dominant fashion with a parent of origin effect. The gene is located on chromosome 11q23. Piccini et al. reported that *SDHD* was the most mutated gene among the genes coding for the SDHX complex (28). Usually, the clinical characteristics of the PGL-1 are multiple head and neck PGLs in patients with an age range of 28–31 years (29). Other tumors such as rare renal cancers and gastric stromal tumors have also been found in these patients (5).

### FPGL-2

The germline loss-of-function mutations in the *SDHAF2 (SDH5)* gene, which is located on chromosome 11q13.1, lead to FPGL-2 (30). This syndrome is transmitted in an autosomal-dominant pattern with a parent of origin effect, similar to *SDHD*. FPGL-2 is commonly associated with parasympathetic PGL at the skull base and neck, and there has been no record of metastases. The average age of initial diagnosis is 33 years, with a range of 22–47 years (31).

### FPGL-3

The gene for FPGL-3 is identified as *SDHC*, which is mapped to chromosome 1q23.3. FPGL-3 is an autosomal-dominant syndrome, which is unlikely to be PCC (32). Most patients develop solitary head and neck FPGLs. Tumors with *SDHC* mutations do not tend to be malignant or multifocal. The mean age of onset for patients with FPGL-3 is 38 years (33).

### FPGL-4

FPGL-4 is caused by inactivating mutations in the tumor suppressor gene *SDHB*, located on chromosome 1p35-36, with an autosomal-dominant inheritance endowing the tumor susceptibility. Clinically, this syndrome is characterized by PGLs in the abdomen, pelvis, and mediastinum (34). The mean age at diagnosis of these tumors is 25–30 years (35). Furthermore, there is an increased risk of malignant PGL associated with this gene mutation (36).

### FPGL-5

The *SDHA* gene mutations that map to chromosome 5p15 can result in FPGL-5. Biallelic mutations in *SDHA* are associated with Leigh’s syndrome (37). *SDHA* germline mutations have been identified in patients with PCC/PGL (38).

### Other mutations

Apart from the typical mutations mentioned above, mutations in *TMEM127* (locus of 2q11.2), *MAX* (14q23.3), *FH* (11q42.1), *SPAS1/HIF2A* (2P21), *EGN1* (1q42.1), and *KIF1β* (1p36.22) have also been proved to be associated with PCC/PGL development (39, 40)*.*

## Genetic Screening Strategies of PCC/PGL

As PCC/PGL accounts for the highest proportion of hereditary-related tumors, it is recommended that all patients with PCC/PGL should be tested for genetic mutations for the following reasons: (i) up to 40% patients carry disease-causing germline mutations (41); (ii) even for sporadic patients, the overall frequency of germline mutation is higher than 10% (42); (iii) specific genetic mutations are related to malignant PCC/PGL; (iv) positive genetic test may lead to an accurate pre-surgery diagnosis; (v) germline mutation in genes may cause other syndromic morbidity; and (vi) positive genetic mutation of the proband may result in an earlier diagnosis of their relatives. To date, approximately 30 different genes have been reported to be related with the morbidity of PCC/PGL and the number is rising with the use of next-generation sequencing (NGS) (6). Thus, using NGS to test the germline mutation of patients with PCC/PGL may be a suitable process. These techniques include whole-genome sequencing (WGS), whole-exome sequencing (WES), and targeted NGS.

### Whole-Genome Sequencing

WGS is a method of sequencing the whole human genome or determining the complete nucleotide sequence of an entire DNA sample (43, 44). It involves sequencing of all the coding and non-coding regions, making it one of the most comprehensive methods (45, 46). With sufficient read depth and fast sequencing time, it has been successfully used to detect chromosomal aberrations in circulating cancer cells (47). WGS has several disadvantages. In order to extract useful data with regard to known disease-causing mutations from enormous data that WGS generate, extensive filtering is usually needed (44). Ethical issues may also be raised when pathogenic mutations are identified in patients who have no known connections to the disease for which the genetic diagnosis was originally requested (44). WGS has been performed to characterize 179 cases of PCC/PGL in order to figure out genomic alterations in PCC/PGL (45, 46). However, high cost and complex data analysis have prevented WGS from being used as a routine diagnostic tool in PCC/PGL (48).

### Whole-Exome Sequencing

Unlike WGS, which analyzes almost all nucleotides of the genome, WES only sequences coding regions of DNA (7). WES allows analysis of all potential disease-causing genes including known disease genes and genes unrelated to a disease (44). WES can also identify new disease genes or mutations that have not yet been associated with certain clinical phenotypes. Compared with WGS, WES can achieve higher read depths at a lower cost. However, regions such as promoters, enhancers, and transcription factor binding sites, which adjoin the exons, are often missed when WES is performed (7). Another limitation of WES is its incomplete representation due to the fact that exons are not included in the manufacturer’s capture design and coverage of base-pair reads in certain exons are relatively low (44). Due to the fact that about 85% of disease-causing mutations are expected to occur within the exome, WES has gained popularity in analysis of PCC/PGL (49). However, when WES is applied in PCC/PGL, the individual exon coverage of PCC/PGL genes and sequence depth are not easily achieved upon the completion of the analysis (7). Also, unsolicited pathogenic mutations are concerns when WES is applied in clinical diagnostic for monogenetic disorders just like WGS.

### Targeted NGS

In targeted NGS method, only the coding regions of genes are enriched to target a specific disease (50). By restricting the mutation detection to a limited gene sets, targeted NGS can achieve better quality of representation and a much higher read depth than WGS or WES. Moreover, as the analysis is targeted at known disease genes, turn-around times for test results are significantly reduced due to simpler procedures in analyzing datasets and interpreting variants (51). In addition, unlike WGS and WES, targeted NGS minimizes the problem of unsolicited findings and thereby increases the willingness of patients in taking part in NGS method (52). In the diagnosis of PCC/PGL, three types of targeted NGS gene panels have been recommended. The first one, the basic panel, includes the PCC/PGL genes mutated at the germline level and is associated with familial disease (7). Second, the extended panel consists of the basic panel genes and candidate genes that have been proven to be functionally relevant in the pathogenesis of PCC/PGL (7). Third, the comprehensive panel includes all the extended panel genes, genes mutated at the germline or somatic level, and the genes found to be exclusively mutated at the somatic level (7, 45, 46). A high degree of diagnostic agreement with targeted NGS and conventional Sanger sequencing method in the analysis of PCC/PGL has been reported in several studies (50, 53). However, the application of targeted NGS is limited by problems like difficulty in adding new genes to existing panels and instruments-based errors.

### Genetic screening according to clinical features and syndromic presentations

NGS is not feasible for all patients because of the high cost. In addition, patients with specific clinical features indicate different germline mutations. Therefore, establishing the priorities for genetic testing with a clinical feature-driven diagnostic algorithm can be a cost-saving and effective screening approach for PCC/PGL patients. Probands or their relatives presented with other syndromic morbidity (Table 1) may be directed for targeted germline mutation testing.

### Genetic screening for patients with bilateral PCC

The presence of bilateral PCC means a relatively high probability of a germline mutation. For such patients, genetic screening of *VHL* and *RET* should be the priority as about 50% of these patients present with *VHL/RET* mutations. In addition, in bilateral PCC, *TMEM127* is mutated in 39.1% of patients and *MAX* is mutated in 66.7% of patients (40, 54). These genes should also be tested for patients without *VHL* or *RET* mutation. Germline mutation of *KIF1Bβ* has also been reported to cause bilateral PCC (55), but it is not recommended for routine test as only a few patients with *KIF1Bβ* mutation have been reported. Although 14.1% of patients with *NF1* germline mutation suffer bilateral PCC (40), the diagnosis of NF1 can be invariably established by clinical findings alone. Thus, genetic screening for these patients may base on syndromic presentations.

### Genetic screening for patients with metastatic PCC/PGL

Because no single histological feature or immunohistochemical profile is able to predict metastatic potential independently, malignancy is established only by the presence of distant metastases in a site where paraganglia are not normally located (56, 57). However, to the modern understanding, PCC/PGL were granted to be malignant as all tumors have metastatic potential. Thus, PCC/PGL should be defined as metastatic or non-metastatic instead of malignant or benign (3). Metastatic disease was reported to occur in 10–20% of PCCs and in 15–35% of abdominal PGLs, and the potential varies depending on the genetic background (58–60). For patients with metastatic disease, *SDHB* should be tested first as more than 40% of this mutation is related to metastatic PCC/PGL and 5.5% of PCC/PGL patients carried *SDHB* germline mutation (27, 40). Further, *SDHD*, *RET*, *VHL*, and *MAX* should be tested if the result of *SDHB* mutation is negative given that 21.4% of PCC/PGL patients carry these germline mutations and those genes were reported to cause metastatic PCC/PGL with a relatively considerable rate (40). Although germline mutation in *FH*, *KMT2D*, and *MEN1* had been reported to cause metastatic PCC/PGL, the proportion of PCC/PGL patients with these mutations is very low (61–63). Therefore, it is not recommended to be a routine test. Although the diagnosis of NF1 can be established by clinical findings, it is reported that 9.3% of patients with PCC/PGL caused by *NF1* germline mutation suffer metastatic disease (40). Thus, genetic screening should be done among PCC/PGL patients clinically diagnosed with NF1 and their relatives for precise and early diagnosis.

### Genetic screening according to the location of the tumor

Although PCC and PGL are both neuroendocrine tumors arising from chromaffin cells and regarded as the same disease, their location and hereditary background are different. Tumors that originate from the adrenal medulla are defined as PCCs, which are thought to be caused by the mutation of *RET*, *VHL*, *MAX*, *NF1,* and TMEM127; while tumors located in extra-adrenal positions are called paragangliomas, which were more likely to be caused by the mutation of *SDHx*, *MDH2,* and *HIF2A* (54). Therefore, genetic screening should also be done according to the location of the tumor.

### Genetic screening according to the genotype–biochemical phenotype relationships

PCC and PGL are neuroendocrine tumors known to generally produce and secrete catecholamines and their metabolites which can be divided into three major phenotypes determined by the underlying molecular pathways of the tumor: (i) noradrenergic phenotype characterized by a (pseudo) hypoxic signature caused by mutations of VHL, SDHx, FH, MDH, and EPAS1; (ii) adrenergic phenotype characterized by activation of kinase signaling pathways caused by mutations of RET, NF1, TMEM127, and MAX; and (iii) dopaminergic phenotype, which is usually seen in patients with metastatic disease caused by SDHB and SDHD mutations. In addition, there are still tumors that do not exhibit elevations in either catecholamines or metanephrines, which is extremely rare, and are called the silent subgroup (64). Given all these, genetic screening could also be performed according to the genotype–biochemical phenotype relationships.

### Genetic screening according to the immunohistochemical features

SDHB immunohistochemistry is negative in tumors mutated on all *SDHx* genes (2). Studies by Papathomas et al. showed that immunohistochemistry for SDHB was a reliable surrogate marker of *SDHx* mutation despite that the SDHB-immunonegative subset of VHL- and NF1-mutated paraganglionic tumors may influence the specificity (65). However, NF1 immunohistochemistry alone does not predict *NF1* gene mutation status in PCCs (66). It is also reported that SDHA is negative only in *SDHA*-mutated tumors, and negative SDHB and SDHA immunohistochemistry on paraffin-embedded tumors can be associated with the presence of *SDHA* mutation. Generally, SDHD immunohistochemistry is positive in tumors mutated on *SDHx* genes. SDHB negative immunostaining is sometimes difficult to interpret because of background staining. So, the addition of SDHD immunohistochemistry will be very useful to predict *SDHx* gene variants in PGL/PCC (67). In addition, study by Korpershoek et al proved high predictive value of negative FH immunohistochemistry for patients with germline *FH* mutations (68, 69). Given these, *SDHx* should be tested for patients with negative SDHB immunohistochemistry, and test of *VHL* and *NF1* should be performed if the test of *SDHx* were negative. *SDHA* should be tested for patients with negative SDHA immunohistochemistry. *FH* should be tested for patients with negative FH immunohistochemistry.

## Conclusion

Genetic screening can be of great importance for all patients with PCC/PGL, and their relatives, especially for those with syndromic manifestation, multiple tumors, metastatic disease, or a young age of onset. Although traditional approach to genetic testing is likely be replaced in the future because of the development of NGS methods, it takes time. Currently, NGS is still unaffordable for many patients with PCC/PGL, and targeted sequencing is more convenient and precise. Given these, preliminary targeted genetic screening can be performed based on clinical features of the patients.
